# Comparable Triglyceride Reduction With Plasma Exchange and Insulin in Acute Pancreatitis – A Randomized Trial

**DOI:** 10.3389/fmed.2022.870067

**Published:** 2022-04-12

**Authors:** Jakob Gubensek, Milena Andonova, Alexander Jerman, Vanja Persic, Barbara Vajdic-Trampuz, Ana Zupunski-Cede, Nejc Sever, Samo Plut

**Affiliations:** ^1^Department of Nephrology, University Medical Center Ljubljana, Ljubljana, Slovenia; ^2^Faculty of Medicine, University of Ljubljana, Ljubljana, Slovenia; ^3^Department of Gastroenterology, University Medical Center Ljubljana, Ljubljana, Slovenia

**Keywords:** acute hypertriglyceridemic pancreatitis, hypertriglyceridemia, apheresis – therapeutic, conservative treatment, free fatty acids

## Abstract

**Background and Aims:**

Both insulin and plasma exchange (PE) are used in hypertriglyceridemic acute pancreatitis (HTG-AP). Our aim was to compare the efficacy of both treatments.

**Methods:**

A randomized, parallel group study performed in a tertiary hospital in 22 HTG-AP patients with non-severe prognosis and triglycerides between 15 and 40 mmol/L. Patients were randomized to daily PE or insulin infusion until triglycerides were <10 mmol/L. Primary outcome was % reduction in triglycerides within 24 h. Secondary outcomes were days needed to lower triglycerides <10 mmol/L, highest CRP and percentage of patients with a severe course of pancreatitis.

**Results:**

There was a trend toward a greater decrease in triglycerides within the first 24 h in the PE group (67 ± 17% vs. 53 ± 17%, *p* = 0.07), but the absolute difference was modest [mean difference of 6 mmol/L (14% of initial value)]. Triglycerides fell below 10 mmol/L in a median (IQR) of 1 (1–2) and 2 (1–2) days, respectively (*p* = 0.25). Secondary outcomes related to disease severity were also comparable: highest CRP 229 vs. 211 mg/L (*p* = 0.69) and severe course of pancreatitis in 2/11 cases in both groups (*p* = 1.0). Regarding treatment complications, there was one mild hypoglycemia and one allergic reaction during PE. Survival was 100% in both groups.

**Conclusion:**

There was no significant difference, but only a trend toward a greater decrease in triglycerides with PE, and the clinical course was also comparable. These results do not support universal use of PE in patients with HTG-AP.

**Clinical Trial Registration:**

[ClinicalTrials.gov], identifier [NCT02622854].

## Introduction

Hypertriglyceridemia is a well-known cause of acute pancreatitis, accounting for 2–10% of cases in the general population (1–3) and up to 48% of cases during pregnancy (4). Although the risk of developing acute pancreatitis statistically increases above a triglyceride level of 2 mmol/L (5), the absolute incidence remains low and levels >10 mmol/L (at which chylomicron formation begins) are usually considered a risk factor for pancreatitis. Pathophysiology of hypertriglyceridemic acute pancreatitis (HTG-AP) is not fully understood. Two mechanisms are probably involved: a) formation of chylomicrons, which increases blood viscosity, causes capillary plugging and leads to ischemia in the pancreas, and b) hydrolysis of triglycerides to free fatty acids (FFA) in the pancreas, which, when they exceed binding capacity of albumin, can cause local damage to acinar cells as well as remote damage to vascular endothelium (2), leading to distant organ damage (2, 6).

There is no clear evidence of a more severe course of HTG-AP compared to acute pancreatitis of other etiologies, with a systematic review concluding that the data are heterogeneous and scarce (7). Furthermore, the level of triglycerides at presentation does not seem to be associated with disease course. Among studies reporting large cohorts, we observed similar triglyceride levels in survivors and non-survivors (8), Hutchison reported no correlation between triglycerides at admission and Ranson score (9) and Zhang found no correlation between triglyceride levels and local or systemic complications (10). However, since triglycerides are directly involved in the pathogenesis of the disease and the persistence of ischemia of the pancreas, rapid reduction of their levels is considered an important treatment goal. In addition to fasting and other conservative measures, several treatments specifically aimed at lowering serum triglycerides have been used: plasma exchange (PE), insulin and heparin.

During PE, patients’ triglyceride-rich plasma is removed by filtration or centrifugation and replaced with a replacement solution, usually a mixture of electrolytes and albumin. The use of PE for removal of triglycerides was first described by Betteridge in 1978 (11). There are moderately sized cohorts published reporting approximately 80% reduction during the first PE or double filtration plasmapheresis (DFPF) treatment (12, 13) and many case series reporting symptomatic relief. On the other hand, less invasive treatment with intravenous insulin and heparin was also described in smaller cohorts. Insulin activates lipoprotein lipase and was successfully used in patients with increased glucose levels (14) or even overt diabetic ketoacidosis, but also in patients without hyperglycemia (15, 16). Reduction of triglycerides of about 44% (17) within the first day was reported in cohorts treated with insulin and 48–72% (9, 13) in cohorts with conservative treatment, often including insulin. There is only one non-randomized comparison of insulin therapy with fasting-only, which did not show a more rapid fall in triglycerides (18). Heparin stimulates the release of endothelial lipoprotein lipase into the circulation, resulting in a transient increase in lipolytic activity followed by a period of decreased lipolysis (19), so its use is no longer recommended (20).

At the time of study design, the use of PE seemed to be the fastest way to lower triglycerides in the setting of HTG-AP. We have previously shown a greater decrease in triglycerides with PE compared to fasting-only periods between PE treatments in an observational study (59% vs. 27% daily, *p* < 0.001) (8), while another small observational study showed no clear clinical benefits of PE over conservative treatment (21). Only recently, some non-randomized comparisons of PE with conservative treatment in small cohorts of patients have been published. Some of them showed a greater reduction of triglycerides with PE (13, 17, 22), while others demonstrated only a comparable reduction (23, 24), and none of the studies showed a clear clinical benefit of PE (13, 17, 21–23). To date, there have been no randomized comparisons between PE and conservative treatments.

Therefore, given the lack of solid data on the efficacy of treatment with PE, we decided to perform a randomized controlled trial comparing triglyceride reduction rates with PE and insulin treatment.

## Materials and Methods

### Study Design

This was a randomized, open-label, parallel group interventional clinical trial performed at a university-affiliated tertiary hospital in patients with presumed mild course of pancreatitis and “moderately” elevated triglycerides. All patients with acute hypertriglyceridemic pancreatitis treated at our institution between June 2016 and July 2020 were screened and included in the study if they met the following criteria: (a) age >18 years, (b) moderately elevated triglycerides between 15 and 40 mmol/L at the first measurement, (c) presumed mild course of pancreatitis at the time of randomization [i.e., no organ failure as defined in the Modified Marshall scoring system (25), which would predict a moderate or severe course of pancreatitis] and the exclusion criterion of pregnancy. Each patient gave a written informed consent prior to randomization. Randomization was not masked and was achieved with a simple randomization list obtained from http://www.randomization.com, using a fixed block randomization method (with a size of 2).

**FIGURE 1:**
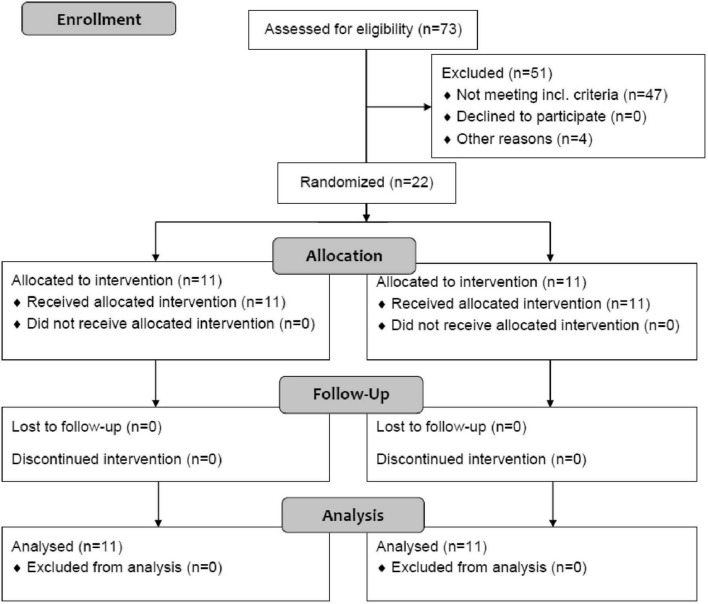
A CONSORT randomized trial flow diagram.

Assuming an average reduction in triglycerides of 65% during the first 24 h with PE [unpublished analysis of first PE treatments in our historic cohort (8)] and 40% with insulin [combined efficacy from two reports of insulin treatment (14, 26)], an alpha error of 0.05, we calculated a sample size of 20 patients for 80% power of the study. For practical reasons, two additional patients, who arrived shortly after the original 20 patients were included, were also randomized for a total number of 22 patients (Figure 1).

The study conforms to the provisions of the Declaration of Helsinki (as revised in 2013) and was approved by the National Medical Ethics Committee (Ref. No. 0120-488/2015-2) and registered at ClinicalTrials.gov (NCT02622854). All authors had access to the study data and reviewed and approved the final manuscript.

### Plasma Exchange and Insulin Treatment

Treatment consisted of nil per mouth, infusions, analgesics and supportive treatment as prescribed by the attending physician. Low molecular weight heparins were used for thromboprophylaxis only at the discretion of the treating physician. Each patient was randomized to receive either daily PE or insulin infusion until triglycerides fell below 10 mmol/L. PE treatment was started as soon as feasible, usually within 12 h after admission, and was performed with membrane technique using regional citrate anticoagulation (27). During each PE, 1 to 1.5 estimated plasma volume was exchanged and replaced with a bicarbonate-based electrolyte solution containing 30 g/L albumin (8). Short-acting insulin was administered intravenously *via* perfusor to patients with elevated glucose (>8 mmol/L) levels, who received 0.9% sodium chloride for volume replacement, whereas patients with normal glucose levels (<8 mmol/L) received 5% dextrose in 0.9% sodium chloride solution with 4 IU of insulin added per 500 mL infused at the discretion of the attending physician. Insulin was adjusted to maintain blood glucose levels between 5 and 8 mmol/L. In case of development of severe course of pancreatitis, a rescue PE was planned. Blood results were taken twice daily (approximately every 12 h) and included serum lipids, C-reactive protein (CRP), electrolytes and other results, as ordered by the attending physician.

### Outcome Measures

The primary outcome measure was the reduction in triglycerides within 24 h after admission, expressed as a percentage of the baseline value. Secondary outcomes were days needed to lower triglycerides below 10 mmol/L, highest CRP level during treatment and percentage of patients with a severe course of pancreatitis. Mortality was also recorded, as were the side effects of treatment (mainly hypocalcemia in the PE group and hypoglycemia in the insulin group). In a subgroup of patients, free fatty acids (FFA) were measured from available residual samples (NEFA kit, Randox, Crumlin, United Kingdom).

### Statistical Analysis

Data are presented as mean ± standard deviation, median and interquartile range (IQR), absolute frequencies or percentages, as appropriate. Statistical analyses were performed using Statistica 12.0 (StatSoft, Inc., Tulsa, United States). Normality of the distribution of variables was checked using Shapiro–Wilk’s W test. Normally distributed continuous variables were compared between groups using Student’s *T*-test and non-normally distributed using Mann-Whitney U test; dichotomous variables were compared using the chi-square test. A *p*-value of <0.05 was considered significant.

## Results

Altogether 22 patients were enrolled in the study and included in final analysis (Figure 1), 11 in each group, and their baseline characteristics are presented in Table 1. All patients received the assigned intervention, were treated according to the protocol and were included in the final analysis. None of the patients in the insulin group received a rescue PE; there were two patients who developed a severe course of pancreatitis after inclusion, but their triglycerides were already <10 mmol/L at 24 h.

**TABLE 1:** Patients’ baseline characteristics and laboratory results in plasma exchange (PE) and insulin group.

Parameter	PE group	Insulin group	*p*-value
N	11	11	/
Age (years)	50 ± 9	52 ± 10	0.73
Male gender	7/11	9/11	0.34
Diabetes	4/11	2/11	0.34
Triglycerides (mmol/L)	31 ± 9	26 ± 8	0.21
Cholesterol (mmol/L)	13.5 ± 4.4	13.3 ± 3.0	0.93
Lipase (μkat/L)	26 ± 25	40 ± 55	0.45
CRP (mg/L)	57 ± 100	55 ± 72	0.95
Creatinine (μmol/L)	70 ± 10	93 ± 74	0.31
Glucose (mmol/L)	11 ± 6	8 ± 3	0.16
Leukocytes (10^9/L)	12 ± 5	13 ± 4	0.50

*Data are presented as mean ± standard deviation or frequencies. To convert TG units to mg/dL, multiply by 88.*

**FIGURE 2:**
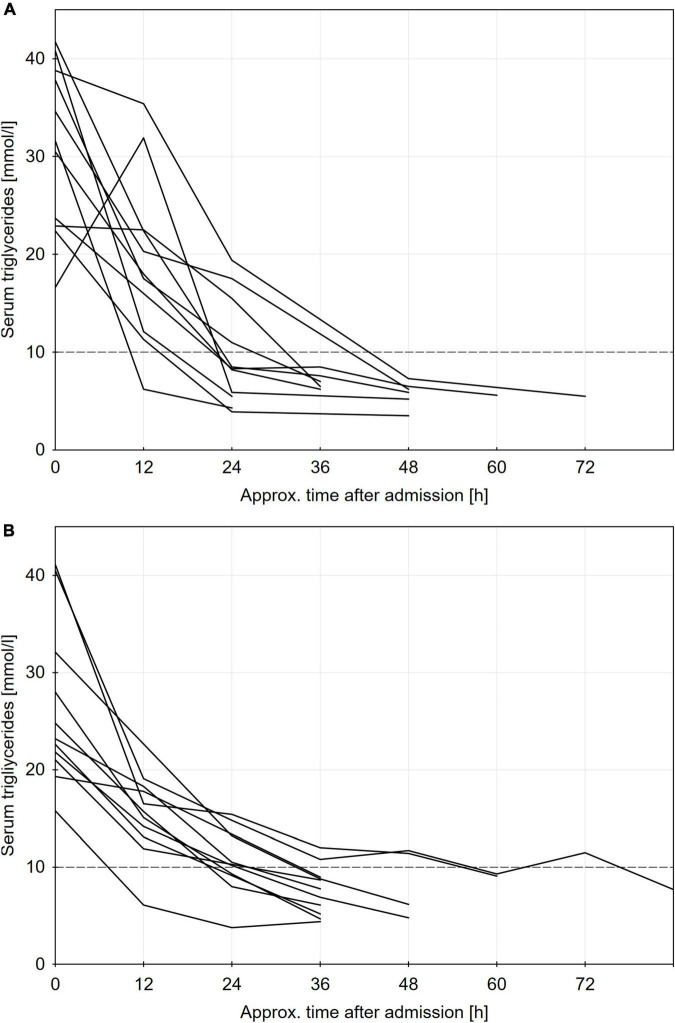
Individual patients triglycerides profiles for plasma exchange panel **(A)** and insulin panel **(B)** groups. To convert TG units to mg/dL, multiply by 88.

Individual patients’ triglyceride level profiles by approximate 12 h time intervals are presented in Figure 2, while the primary and secondary outcomes are presented in Table 2. There was a trend toward a greater decrease in triglycerides within 24 h after admission in the PE group (67 ± 17% vs. 53 ± 17%, *p* = 0.07), but the absolute difference in treatment efficacy was modest (mean difference of 6 mmol/L (95% CI −1 to 15 mmol/L) within 24 h or 14% (95% CI 0 – 28%) of the baseline triglycerides). In parallel, cholesterol levels were also borderline lower after 24 h in the PE group. Regarding secondary outcomes, triglycerides fell <10 mmol/L at median after 1 (IQR 1–2) day in the PE group and after 2 (1–2) days in the insulin group (*p* = 0.25). In a subgroup of patients, FFA were comparable within 12 h of admission, and reduced to similar values after 24 h (Table 2).

**TABLE 2:** Triglyceride (TG), cholesterol, free fatty acids (FFA) levels and main clinical outcomes in plasma exchange (PE) and insulin group.

Parameter	PE group	Insulin group	*p*-value
Admission TG (mmol/L)	31 ± 9	26 ± 8	0.21
TG at 12 h (mmol/L)	20 ± 9	15 ± 4	0.12
TG at 24 h (mmol/L)	10 ± 5	12 ± 3	0.31
Reduction in TG at 24 h (mmol/L)	21 ± 9	15 ± 7	0.07
% reduction in TG at 24 h	67 ± 17%	53 ± 17%	0.07
Days until TG <10 mmol/L	1 (1 − 2)	2 (1 − 2)	0.25
Cholesterol at 24 h (mmol/L)	4.8 ± 1.7	8.8 ± 3.3	0.05
FFA within 12 h of admission (mmol/L)	5.3 ± 1.3^a^	5.3 ± 2.2^b^	1.00
FFA at 24 h (mmol/L)	2.0 ± 1.5^a^	2.7 ± 2.9^b^	0.57
Highest CRP (mg/L)	230 ± 94	211 ± 119	0.16
Severe course of pancreatitis	2/11	2/11	1.0
Hospital stay (days)	9 (7 − 31)	9 (5 − 16)	0.29
Mortality	0%	0%	/

*^a^N = 7.*

*^b^N = 5. Data are presented as mean ± standard deviation, frequencies or median and inter-quartile range. To convert TG units to mg/dL, multiply by 88.*

The severity of pancreatitis was comparable in both groups; a severe course developed in 2 cases in both groups and the highest CRP was also comparable (229 vs. 211 mg/L, *p* = 0.69). Hospital length of stay was also comparable in both groups and survival was 100%.

Regarding complications of the two triglyceride-lowering treatments, there was one mild hypoglycemia in the insulin group and one allergic reaction (urticaria and hypotension) in the PE group, occurring toward the end of the PE procedure and led to premature discontinuation of the PE procedure, which was probably unrelated to the PE because albumin was used as a replacement solution.

## Discussion

To our knowledge, this is the first randomized trial, comparing the efficacy of PE and insulin treatment in lowering of serum triglyceride levels in patients with HGT-AP. We found a trend toward a modestly greater decrease in triglycerides within 24 h with PE treatment and a comparable clinical course of pancreatitis (although the study is underpowered for such comparison). These data undermine the assumption that PE lowers triglycerides more rapidly than conservative treatment, which is often considered a surrogate treatment goal, aimed at alleviating the course of HTG-AP.

Plasma exchange has been used for decades in some centers with the aim of a rapidly lowering triglyceride levels in patients with HTG-AP. Current apheresis guidelines state that the optimal role of apheresis in the treatment of HTG-AP has not been established and suggest individualized approach (28, 29). Seeing a milky, lipemic plasma removed from the patient and with a reported reduction in triglycerides of up to 80% within the first 24 h (12, 13), PE seems a very reasonable treatment.

There were rare attempts in the literature at a controlled comparison of PE with conservative treatments, showing greater triglyceride lowering with PE in some (8, 13, 17, 22) but not all reports (23, 24). E.g., we have shown a 59% vs. 27% daily reduction in triglycerides with PE as compared to fasting-only 24-hour periods in between PE treatments (8), with the limitation, that the fasting periods usually did not occur on the day of admission and that the% reduction in triglycerides is likely higher with higher baseline triglyceride level. Another retrospective study found a significantly higher baseline triglycerides in the apheresis group and comparable levels after 24 h, resulting in a 79% decrease with apheresis versus 44% with insulin infusion (17). In a relatively large propensity score-matched cohort of patients, a modest but significantly greater reduction in triglycerides was seen with DFPF (80%) versus conservative treatment (72%), which included insulin only in cases of hyperglycemia (13); a result comparable to this study. In another controlled study with fasting only (insulin given only for hyperglycemia), a 48% reduction after 24 h was achieved, compared to 71% in the PE group (22). In contrast, two other studies showed no effect of PE on the time course of triglycerides in a cohort of approximately 30 patients with HTG-AP (23, 24). It should be acknowledged that focusing on the first 24 h of what could be considered as an “emergency” treatment is perhaps clinically more relevant and also has greater statistical power than looking at an overall (over several days) course of triglyceride levels in two groups. Furthermore, large cohorts treated conservatively without PE, have recently been reported with a median reduction in triglycerides of 48% (IQR 29–63%) within the first 24 h and comparable clinical outcomes (median hospital stay of 6 days and mortality of 1.7%) to the published PE cohorts (9). Data from the literature therefore suggest a mildly higher efficacy of PE compared to conservative treatment, and our results are consistent with this, showing a statistically borderline and probably clinically irrelevant difference in the efficacy of both treatments.

As noted above, recently published large conservatively treated cohorts show significant and comparable triglyceride lowering with fasting alone (13, 22) or with an addition of insulin (9). Therefore, fasting, fluid replacement and endogenous triglyceride metabolism likely achieve the majority of the fall in triglycerides and are therefore the mainstay of treatment. It should be noted that insulin treatment has also not yet been compared to fasting alone in a randomized trial, but such a trial is underway (30). A controlled study comparing both treatments also did not show intravenous insulin to result in a more rapid decrease in triglycerides compared to fasting alone (18). Therefore, it is likely that fasting alone would achieve similar results, with the possible exception of patients with severe triglyceride metabolism disorders.

The validity of a fast reduction of triglycerides itself as a surrogate treatment goal could also be questioned, as the final goal of treatment is amelioration of the severity of pancreatitis. Data on the effect of PE on the clinical course of pancreatitis are even more scarce, but none show a convincing benefit of PE (21–23), not event in studies, where greater reduction of triglycerides was observed with PE (13, 17). Several large randomized trials are currently underway to address the issue of hard clinical end-points in the treatment of HTG-AP with either PE, insulin or fasting alone, which will provide more definitive data in the next few years (30, 31).

Finally, PE could have beneficial effects on the course of acute pancreatitis independently of the triglyceride-lowering effect itself. PE (but not DFPF, which reinfuses the small molecules) can remove inflammatory mediators and other mediators of distant organ failure, e.g., FFA (32). For example, in patients with septic shock PE was shown in some studies to improve hemodynamic stability and reduce vasopressor dose (33), so it could be beneficial in reducing the systemic inflammatory response syndrome in HTG-AP. Additionally, FFA are released from the pancreas and surrounding fat tissue and are one of the mediators of distant organ failure in HTG-AP (6) and also in acute pancreatitis of other etiologies (34). FFA levels were shown to be higher in necrotizing pancreatitis (35, 36) and are likely responsible for a more severe course of pancreatitis in obese patients (37). Our preliminary data from a small subgroup of patients show comparable reduction in FFA levels with both treatment groups, which therefore does not support the use of PE with this aim.

Limitations of the study include its relatively small size, open-label design (blinded studies in the field of apheresis are virtually non-existent), exclusion of patients with a predicted severe course of pancreatitis and using a simple method of randomization.

## Conclusion

In this first randomized comparison with insulin infusion, there was no difference in the decrease in triglycerides within the first 24 h after admission. There was only a trend toward a greater decrease with PE treatment and the absolute difference between the groups was modest, probably clinically irrelevant and in our opinion does not justify the cost and invasiveness of treatment with PE. The clinical course of pancreatitis was comparable, although the study was not powered to detect such differences. The results of this study do not support the universal use of PE in patients with hypertriglyceridemic pancreatitis and further studies are necessary to elucidate its potential beneficial effects in a subgroup of patients with a suboptimal lowering of triglycerides with conservative treatment or patients with a severe course of pancreatitis.

## Data Availability Statement

The raw data supporting the conclusions of this article will be made available by the authors, without undue reservation.

## Ethics Statement

The studies involving human participants were reviewed and approved by National Medical Ethics Committee. The patients/participants provided their written informed consent to participate in this study.

## Author Contributions

JG, MA, AJ, VP, BV-T, AZ-C, NS, and SP performed the research. JG and SP designed the study and collected and analyzed the data. JG wrote the manuscript. All authors reviewed and approved the final version of the article.

## Conflict of Interest

The authors declare that the research was conducted in the absence of any commercial or financial relationships that could be construed as a potential conflict of interest.

## Publisher’s Note

All claims expressed in this article are solely those of the authors and do not necessarily represent those of their affiliated organizations, or those of the publisher, the editors and the reviewers. Any product that may be evaluated in this article, or claim that may be made by its manufacturer, is not guaranteed or endorsed by the publisher.
